# Supplementary material for: Systematic comparison of sea urchin and sea star developmental gene regulatory networks explains how novelty is incorporated in early development
Source: Nat Commun. 2020 Dec 4;11:6235. doi: 10.1038/s41467-020-20023-4 (PMC7719182; doi:10.1038/s41467-020-20023-4)
Supplement: Supplementary file 1 — Supplementary Information [file 41467_2020_20023_MOESM1_ESM.pdf]

Supplementary Information File for:

Title:

Systematic comparison of sea urchin and sea star developmental GRNs explains how novelty is incorporated in early development

Authors:

Gregory A. Cary[1,2], Brenna S. McCauley[1,3], Olga Zueva[1], Joseph Pattinato[1], William Longabaugh[4], Veronica F. Hinman[1,*]

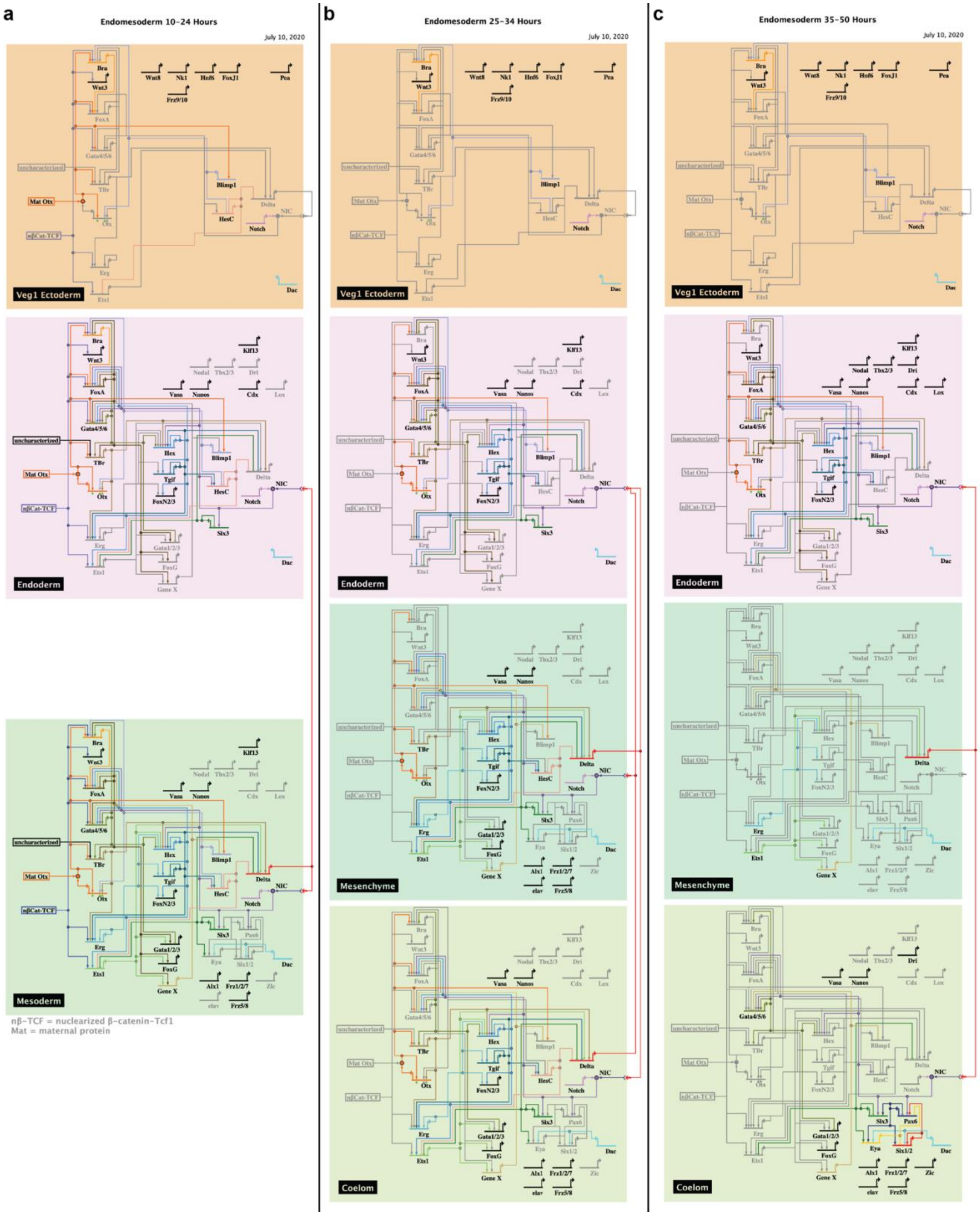
a
Endomesoderm 10-24 Hours
July 10, 2020
Veg1 Ectoderm
Endoderm
Mesoderm
b
Endomesoderm 25-34 Hours
July 10, 2020
Veg1 Ectoderm
Endoderm
Mesenchyme
Coelom
c
Endomesoderm 35-50 Hours
July 10, 2020
Veg1 Ectoderm
Endoderm
Mesenchyme
Coelom
nβ-TCF = nuclearized β-catenin-Tcf1
Mat = maternal protein

Supplementary Figure 1. ***P. miniata*** **endomesodermal network through 50 hours of embryogenesis.** These images represent screenshots of the summary views of the extended network available online (grns.biotapestry.org/PmEndomes). The nodes of the network are genes and the horizontal lines represent cis-regulatory input regions into each gene. The nodes are connected by edges that indicate either positive (arrow) or repressive (bar) relationships between two specific genes. Edges with experimental cis-regulatory support are indicated (green diamonds). Genes are grouped into territories (colored boxes) in which they are expressed during specific developmental stages. Signaling between cell types is indicated as double arrow heads. The expression and regulatory linkages are included here as reported and have been ordered according to the embryonic chronology and spatially arranged into appropriate territories. There are three principal temporal subdivisions that span 10-24 hpf (A), 25-34 hpf (B), and 35-50 hpf (C), the breakpoints relating to major embryonic milestones, *i.e.* the distinction of mesoderm from endoderm at ~24 hpf and subsequently the split between mesenchymal and coelomic fated mesoderm at ~35 hpf. An extended, interactive version with dynamic, hour-by-hour views of embryonic progression as well as detailed information about experimental evidence supporting each node and edge assignment is available (grns.biotapestry.org/PmEndomes). The experimental data supporting the construction of this model is available as a supplemental file to this manuscript (Supplementary Data, .xlsx format) and the network itself is provided as a separate supplemental data file (Supplementary Software, .btp format).

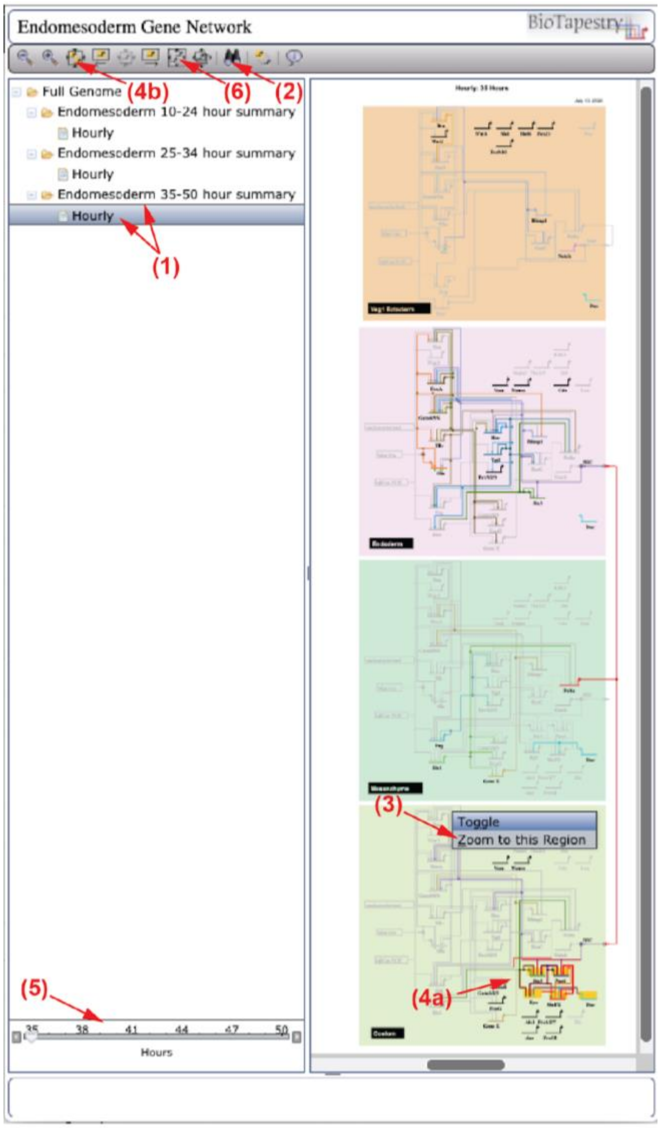

Supplementary Figure 2. Navigation through the online version of the GRN model. Navigating the model, available at grns.biotapestry.org/PmEndomes, involves selection of either the summary view or the hourly view (1) of a specific submodel. Genes within the submodel can be identified by searching (2, binocular icon). Higher resolution views of each region can be accessed by zooming to a particular region (3) or highlighting groups of genes (4a) and magnifying the highlighted nodes (4b). The temporal aspect of hourly models can be examined using the time slider (5). Returning to the entire sub-model view can be achieved by clicking on the icon indicated (6). To access information about the experimental data supporting each node or edge, one can click on a network element and right click to bring up a contextual menu from which one can select “Experimental Data”.

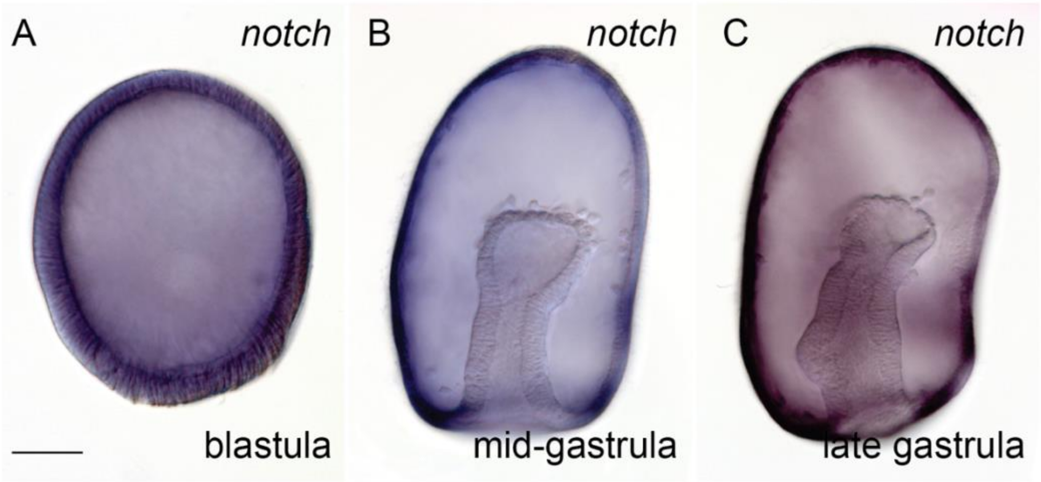

Supplementary Figure 3. Expression of the sea star *notch* transcript by colorimetric WMISH. RNA encoding the Notch receptor is ubiquitously distributed at blastula stage (A) and is restricted to ectodermal expression at mid- and late-gastrula staged (B,C). Data are representative of two biologically independent experiments consisting of at least 10 embryos each. Scale bar represents 50 μm; applicable to all images in the panel.

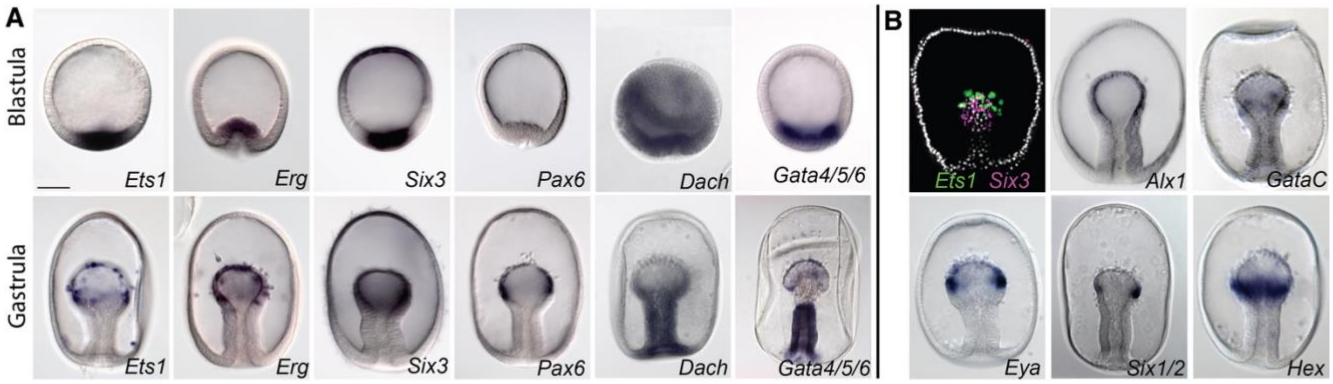

Supplementary Figure 4. Molecularly uniform vegetal pole mesoderm partitions into blastocoelar mesenchyme and coelomic epithelium by mid-gastrula stage. (A) Colorimetric WMISH staining of *ets1*, *erg*, *six3*, *dach,* and *gata4/5/6* transcripts show that they are all expressed in the vegetal pole territory in blastula stage embryos in overlapping domains. The *gata4/5/6* transcript is distributed slightly broader owing to the fact that it is also expressed in the endoderm and the *dach* transcript is expressed ubiquitously early. Expression of *pax6* is not detected until later stages. By mid-gastrula stage (bottom row) both *ets1* and *erg* are expressed in ingressing mesenchyme cells, while *six3, pax6, dach,* and *gata4/5/6* are all expressed in the mesodermal epithelium at the top of the archenteron. Both *dach* and *gata4/5/6* are also both expressed throughout the endoderm at this stage. Additional coelomic mesoderm genes are expressed in the posterior aspect of the mesodermal bulb of the archenteron by mid-gastrula stage (B). The expression of *eya*, *six1/2, alx1*, and *hex* transcripts are all co-localized in this territory. Importantly, as shown by dFISH, *ets1* expressing cells are distinct from *six3* expressing cells at this stage. Data are representative of two biologically independent experiments consisting of at least 10 embryos each. Scale bar represents 50 μm; applicable to all images in the panel.

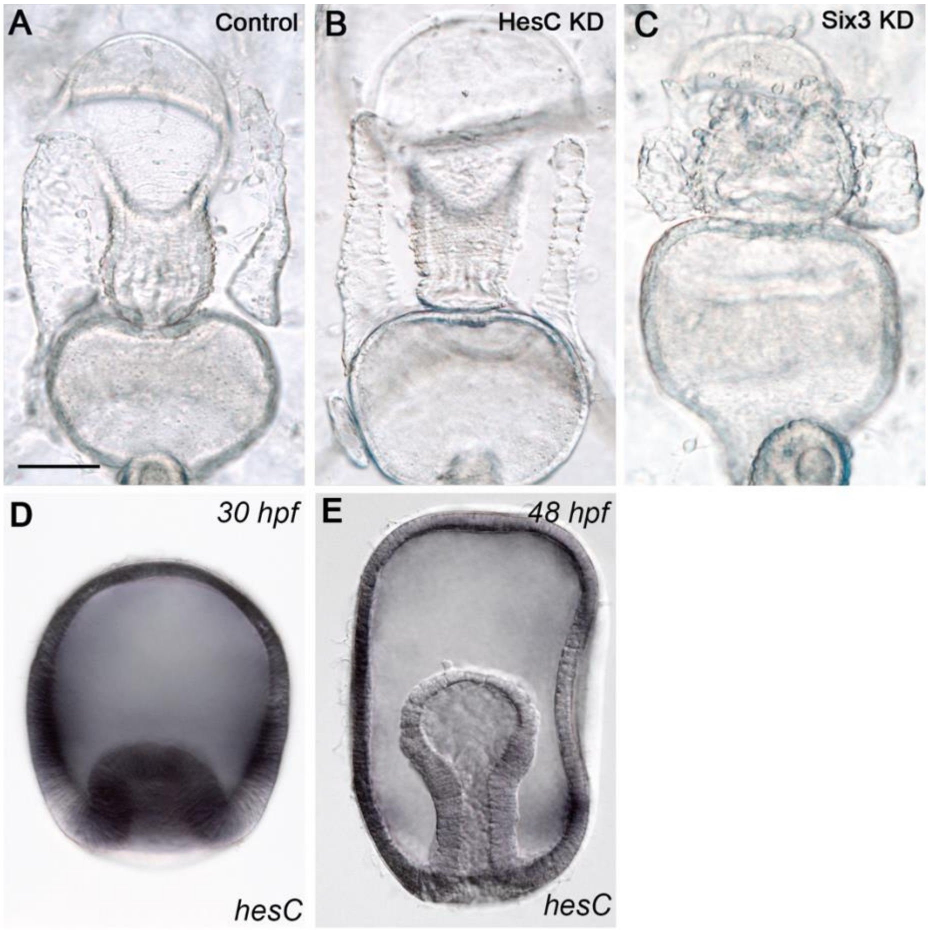

Supplementary Figure 5. HesC handoff by 48 hpf. MASOs targeting *hesC* do not perturb the morphology of the coelomic epithelium at 6 days post fertilization (A, B), while perturbation of *six3* leads to much diminished coelomic epithelium as well as a shortened foregut (C). The expression of *hesC* transcript, detected in early mesodermal territories by colorimetric WMISH (D), is undetectable in mesoderm by 48 hpf (E). Data are representative of two biologically independent experiments consisting of at least 10 embryos each. Scale bar represents 50 μm; applicable to all images in the panel.

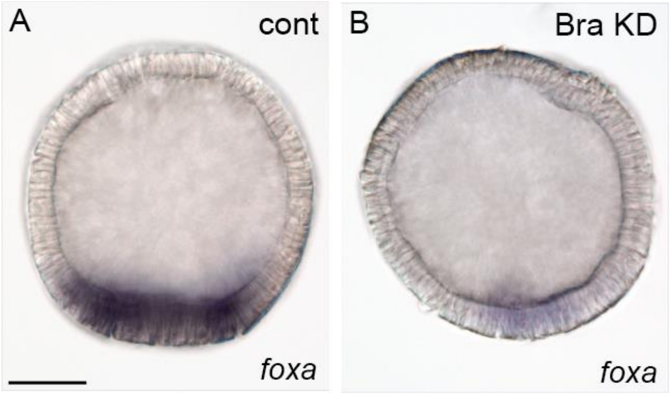

Supplementary Figure 6. *foxa* responsive to Bra knockdown. Knockdown of *foxA* transcript by morpholino oligonucleotide targeting the Bra transcript (B) compared to control morpholino injection (A) by colorimetric WMISH. Data are representative of two biologically independent experiments consisting of at least 10 embryos each. Scale bar represents 50 μm; applicable to all images in the panel.

Supplementary Table 1. Morpholino antisense oligonucleotide sequences and concentrations used.

Morpholino	Sequence	Injection Solution Concentration
Tgif_1	TCGAGAGCCGATCCACGAATCAAGA	800 µM
Tgif_2	TGCAACAACAAAGACGGTTCGGTAC	400 µM
HesC_1	CAATCATGCTGAAGATTGTCGAAGG	600 µM
HesC_2	TGTTCCGAGTAGAAGACGAATCGA	600 µM
Six3_1	ACATTGAGCCGAGCATCTGGACCCG	600 µM
Six3_2	TCTCAGCAGCGCAGTCGAGAGACAC	600 µM
Pax6_1	AAGTGCTTCACTGACCTGTATCCTA	400 µM
Pax6_2	CTCTGAAGTAAACTGTGTATAAGGC	600 µM
Eya_1	GCACGTAGTTGAAGCAAACACATCA	600 µM
Six1/2_1	CGTGAAGCCAAACGACGGCAACATG	600 µM
Krox_1	CAGGTCCTTTCATTCTGGTACTCAG	600 µM
Bra_1	CACTCATGGTGTTTCAAAAATGCTC	600 µM
Standard Control Oligo	CCTCTTACCTCAGTTACAATTTATA	600 µM

Supplementary Table 2. Primers used for quantitative RT-PCR assessment of knockdown and drug treatment conditions.

Gene	F	R
Hesc	AAGCCTCATCTTCCCAGCTCTC	CCTTCAGGTAACGGACGGTCAT
Delta	CGAAGGCTTCACGTGCTACTG	GCGCATGCGTAGCCATTCTC
lamin2b receptor	GAGCATGCCTAAGCCAGACC	CTCCACCATGGGCTCCAGTA

Supplementary Table 3. Descriptions and phenotype counts for morpholinos to coelomic network genes.

Morpholino	WMISH Probe	Phenotype	# Counted	# Phenotypic	% phenotypic
Pax6	Eya	Decrease	37	30	81.1%
Six3	Eya	Decrease	37	27	73.0%
Pax6	GataE	no mesodermal expression	48	4	8.3%
Eya	GataE	no mesodermal expression	45	34	75.6%
Six1/2	GataE	no mesodermal expression	44	20	45.5%
Eya	Pax6	clearance loss	46	40	87.0%
Pax6	Pax6	clearance loss	128	81	63.3%
Six1/2	Pax6	Decrease	37	21	56.8%
Pax6	Six1/2	Decrease	89	66	74.2%
Eya	Six3	normal expr	28	28	100.0%
Pax6	Six3	Off in anterior mesoderm	47	21	44.7%
Six1/2	Six3	normal expr	95	75	78.9%
Six3	Six3	Increase	7	7	100.0%
Pax6	-ALL-	split archenteron	174	26	14.9%
