# Supplementary material for: Systematic comparison of sea urchin and sea star developmental gene regulatory networks explains how novelty is incorporated in early development
Source: Nat Commun. 2020 Dec 4;11:6235. doi: 10.1038/s41467-020-20023-4 (PMC7719182; doi:10.1038/s41467-020-20023-4)
Supplement: Supplementary file 6 — Description of Additional Supplementary Files [file 41467_2020_20023_MOESM6_ESM.pdf]

Title: Supplementary Data 1.
Description: Summary of experimental evidence supporting node expression and edge connections in the sea star endomesodermal network. Summary of experimental evidence supporting node expression and edge connections in the Patiria miniata endomesodermal GRN

Title: Supplementary Data 2.
Description: Biotapestry file for Patiria miniata endomesodermal GRN
